# GOtcha: a new method for prediction of protein function assessed by the annotation of seven genomes

**DOI:** 10.1186/1471-2105-5-178

**Published:** 2004-11-18

**Authors:** David MA Martin, Matthew Berriman, Geoffrey J Barton

**Affiliations:** 1 Post-Genomics and Molecular Interactions Centre, School of Life Sciences, University of Dundee, Dow Street, Dundee DD1 5EH, UK; 2 The Sanger Institute, Wellcome Trust Genome Campus, Hinxton, Cambs CB10 1SA, UK

## Abstract

**Background:**

The function of a novel gene product is typically predicted by transitive assignment of annotation from similar sequences. We describe a novel method, GOtcha, for predicting gene product function by annotation with Gene Ontology (GO) terms. GOtcha predicts GO term associations with term-specific probability (P-score) measures of confidence. Term-specific probabilities are a novel feature of GOtcha and allow the identification of conflicts or uncertainty in annotation.

**Results:**

The GOtcha method was applied to the recently sequenced genome for *Plasmodium falciparum *and six other genomes. GOtcha was compared quantitatively for retrieval of assigned GO terms against direct transitive assignment from the highest scoring annotated BLAST search hit (TOPBLAST). GOtcha exploits information deep into the 'twilight zone' of similarity search matches, making use of much information that is otherwise discarded by more simplistic approaches.

At a P-score cutoff of 50%, GOtcha provided 60% better recovery of annotation terms and 20% higher selectivity than annotation with TOPBLAST at an *E*-value cutoff of 10^-4^.

**Conclusions:**

The GOtcha method is a useful tool for genome annotators. It has identified both errors and omissions in the original *Plasmodium falciparum *annotation and is being adopted by many other genome sequencing projects.

## Background

It is now often possible to obtain the complete genome sequence of an organism in a few months, but without a directed approach, determining the function of potential gene products through biological experimentation is inefficient. Accordingly, methods for function prediction are required to direct experiments in function verification. In the context of this paper the term function is used to refer to all aspects of a gene product's behaviour. This includes the concepts described by the Gene Ontology classifications for Molecular Function, Biological Process and Cellular Component. It is explicitly stated in the text when a more specific interpretation of function is intended.

A powerful tool in the annotation of novel genomes is the prediction of function by similarity to a sequence of known function. Such 'transitive function assignment' can work very well where there is a clear match to a homologue with a well established function. However, accurate functional assignment is difficult in cases where the match is less well defined, either due to lower sequence similarity or the presence of many candidates with differing functions. Gerlt and Babbitt [1] reviewed a number of examples where sequence similarity alone cannot provide full function specificity. The examples they discussed included classes of proteins where the function is similar but sequences are diverse, and classes where sequences are similar but function is diverse, indicating potential pitfalls for automated analyses. These examples are however quite extreme; sequence similarity can be used to infer function for a large proportion of genes with good results. Function annotation of sequences by tools such as PEDANT [2] and GeneQuiz [3] was dependent on free text annotations in the sequence databases and was complicated by the difficulty of mining and interpreting natural language. For example, a function may be described in one way in one sequence annotation, only to have the same function described in a different way in another sequence annotation. Such inconsistencies make computational determination of function equivalence difficult if not impossible. The use of restricted vocabularies and keywords has gone some way towards addressing this problem since it allows direct comparison of sequences with identical annotation schemes, at least to a match/no match level. Ouzounis and Karp [4] proposed the Transitive Annotation Based Score (TABS) to assess qualitatively the differences between annotations provided by different schemes. This scale relies on a human curator to determine manually the relationship between potentially conflicting terms, so is not readily applicable to the automated analysis of annotations.

Keywords and restricted vocabularies do not solve the problem of conflicting assignments. Unless some computable form of relationship is present between terms, it is not possible to provide any automated form of conflict resolution between terms or to identify computationally where one term is a more specific descriptor than another.

An ontology represented as a graph can provide a solution to this problem. Ontologies are restricted vocabularies, or sets of terms where each term is explicitly related to parent terms and child terms (and hence to sibling terms). The Gene Ontology (GO) [5] is a description of biology represented as a directed acyclic graph (DAG) where each node represents a clearly defined biological concept. Gene Ontology is continually being developed but contained approximately 14,000 nodes as at March 2003.

The availability of the Gene Ontology has provided for the first time, a broadly accepted classification system for function assignment that can be analysed computationally. Previous work using other classification schemes, such as restricted vocabularies based on SwissProt keywords, suffered because of the lack of a distinct relationship between terms and/or due to typographical differences [6,7]. Since the establishment of GO, several authors have prepared tools that provide function assignment to Gene Ontology or a subset thereof. Jensen and co-workers [8] used neural networks to provide predictors for a small subset of 190 relatively non-specific GO terms. Schug et al. [9] used similarity to protein families defined as ProDom [10] or CDD [11] domains, by assigning the most specific common function represented in the set of proteins belonging to the family. This was a relatively conservative approach, taking similarity to clearly defined families annotated with relatively non-specific functions as a basis for transitive annotation.

Xie and coworkers [12] have combined sequence similarity data with protein domain matches, cellular location prediction and literature mining data to improve transitive assignments. Their tools provided mappings to individual GO terms using a complex collection of probabalistic models and single linkage clustering. The method appears to be extremely powerful, taking input from a wide variety of sources, but it is difficult to assess the overall accuracy.

Two tools based on BLAST searches have recently been presented in the literature. OntoBlast [13] provided a list of GO-terms prepared from gene-association links to similarity matches from BLAST searches. GO terms associated with the matching sequences are scored according to the *E*-value of the pairwise match. GOblet [14] also applies BLAST searches as the basis for assigning GO terms but does not give any estimates of validity beyond restricting matches to those below a user defined E-value threshold and counting the number of matching sequences.

In this paper we present a novel method, GOtcha, that can be applied to any database search technique that returns scored matches. We have initially implemented this with BLAST searches and extend the analysis from the similarity match scores for a search in order to provide an empirical estimate of the confidence in each predicted function. We have applied this method to Malaria (*Plasmodium falciparum*) [15] and six other well annotated genomes and compared the results obtained by the GOtcha method to the results of annotation with the top informative BLAST match. The two methods have been assessed quantitatively with seven-fold cross validation by comparing the predictions obtained by GOtcha with those provided by the curators of the respective genome sequence consortia.

The assessment of the global accuracy of a particular annotation method is extremely problematic in the absence of a computable annotation scheme. Gene Ontology provides such a computable scheme and we present here a quantitative measure for comparison of function annotations based on assignment to GO terms. This provides a metric for direct objective comparison of annotation methods that is independent of arbitrary cut off values. The new accuracy measure encompasses true positives, false positives and false negatives, so combining sensitivity and selectivity in one value.

## Results

Two sets of annotation predictions were determined for each data set in the study. One was based on all available GO annotations and the other on a reduced set of GO annotations that excluded gene-associations with the evidence code IEA (Inferred by Electronic Annotation). IEA annotations are usually considered to be less reliable as they have not been assessed by a human curator. In contrast, ISS annotations (Inferred from Sequence Similarity) are annotations which, whilst being derived electronically, have been assessed by a human curator and can be considered sufficiently reliable. IEA annotations may however give a broader coverage than non-IEA annotations. On average, each dataset contained slightly more than 50% IEA annotations, though the vast majority of the sequences had some non-IEA annotation. The number of sequences for each dataset is listed in Table 1 along with a summary of the number of sequences annotated both with and without IEA annotations.

### Function assignment using all gene-associations

**Figure 1:**
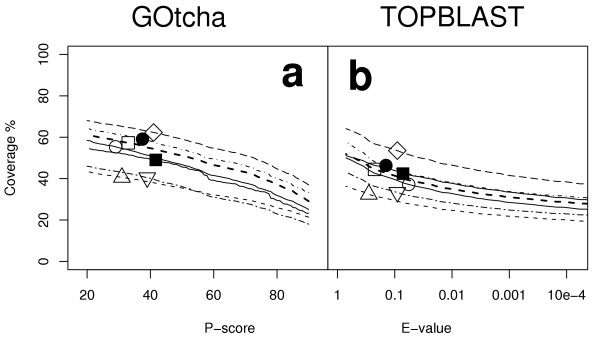
**Proportion of original GO annotations recovered versus cutoff for assignment of GO terms. **(a) GOtcha (b) top informative BLAST hit (TOPBLAST). For GOtcha the P-score is defined in the text. For TOPBLAST the *E*-value is the expectancy score for the top annotated sequence match.Key: ○ *Arabidopsis thaliana*; △ *Drosophila melanogaster*; □ *Homo sapiens*; ● *Plasmodium falciparum*; ■ *Vibrio cholerae*; ◇ *Caenorhabditis elegans*; ▽ *Saccharomyces cerevisiae*.

Figure 1 illustrates the recovery of annotations by the two function assignment methods. In this and the following analyses the predicted term associations for all three ontologies are combined. The derivation of the P-score accuracy estimate (see Methods) normalises the data allowing combination of the three separate sets of results in one graph. The *y*-axis indicates the proportion of annotations provided by the genome project (given annotations) that were annotated to some degree by either GOtcha (Figure 1a) or TOPBLAST (Figure 1b). At a P-score of 50% GOtcha recovered 47% of the given annotations (35–59% s.d. 7.7%) whereas TOPBLAST with a cutoff of *E *= 10^-4 ^recovered 28% of annotations (20–38% s.d. 5.1%). This *E*-value cutoff is at the top end of the *E*-values between 10^-4 ^and 10^-20 ^typically used as a threshold for confident function assignment [16-20]. The proportion of annotations recovered by TOPBLAST was on average 60% (s.d. 4.1) of the proportion of annotations recovered by GOtcha, clearly indicating the presence of much useful function information throughout the BLAST search results, even at relatively high *E*-values.

**Figure 2:**
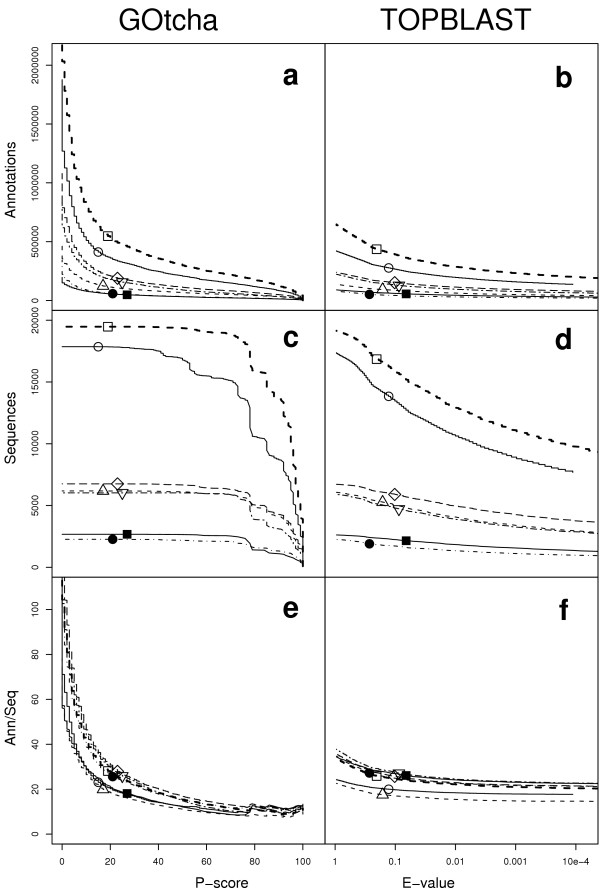
**Annotations and sequences annotated. **Number of GO term associations made by (a) GOtcha with a P-score over the cutoff and (b) TOPBLAST with an *E*-value below the cutoff. Number of sequences with an associated annotation predicted by (c) GOtcha with a P-score over the cutoff and (d) TOPBLAST with an *E*-value below the cutoff. P-score iscalculated to 1 percentage point resolution giving rise to the stepped nature of the graph. Mean number of annotations per annotated sequence predicted by (e) GOtcha and (f) TOPBLAST. Key: ○ *Arabidopsis thaliana*; △ *Drosophila melanogaster*; □ *Homo sapiens*; ● *Plasmodium falciparum*; ■ *Vibrio cholerae*; ◇ *Caenorhabditis elegans*; ▽ *Saccharomyces cerevisiae*.

Figure 2 illustrates for each genome the total number of predicted GO term associations (GOtcha in Figure 2a, TOPBLAST in Figure 2b) and the number of sequences annotated (GOtcha in Figure 2c, TOPBLAST in Figure 2d) with respect to a scoring cut off for the annotation by each method. Figure 2e (GOtcha) and Figure 2f (TOPBLAST) illustrate the number of annotations per annotated sequence. Figures 2a,2c and 2e show the results for GOtcha with the *x*-axis representing the minimum P-score. A low P-score represents low confidence in the annotation. A high P-score represents high confidence in the annotation. Figures 2b,2d and 2f show the results for the top informative BLAST hit with the *x*-axis representing the maximum *E*-value. A low *E*-value represents high confidence in the annotation. A high *E*-value represents low confidence in the annotation. In Figure 2c the total number of sequences annotated by GOtcha with a P-score for the annotation above the value on the *x*-axis approaches the maximum relatively quickly when moving from high P-score to low P-score, typically coming very close to the total number of sequences annotated well before the P-score has dropped to 50%. This represents a broad coverage of sequence space, assigning annotation at a relatively nonspecific level to most sequences. In terms of the total number of annotations, these rise steadily as the P-score cut off drops. At very low P-scores (below 10%) the total number of annotations increases rapidly, indicating an increase in the spectrum of functions matched with only weak similarity. The number of annotations per sequence increases gradually as the P-score drops until a rapid rise at low P-scores (Figure 2e). The rapid increase in number of sequences annotated is a reflection of high confidence in GO term associations at a general level of specificity. At lower P-score values more specific terms can be associated with sequences but the total number of sequences annotated has already approached the maximum. In comparison, the average number of associated GO terms per sequence by the genome projects varies from 14.5 to 19.8 (mean 16.6 s.d. 1.9). Figure 2d shows the number of sequences annotated using the top BLAST hit with a score below the *E*-value indicated by the *x*-axis. In this case the number of sequences annotated increases more slowly with *E*-value (Figure 2d) but the number of annotations per sequence remains relatively constant, rising only modestly as *E*-value rises (Figure 2f). This arises from the key difference between GOtcha and TOPBLAST. In GOtcha a term-specific probability is calculated which allows some functions for a given sequence to be assigned more confidently than others. For a given sequence only the more general terms will appear in the prediction list above the P-value threshold. With TOPBLAST the whole set of annotations from the top matching hit is assigned with a common score, irrespective of the term's specificity. Thus either all or no terms for that sequence will appear below the *E*-value threshold.

**Figure 3:**
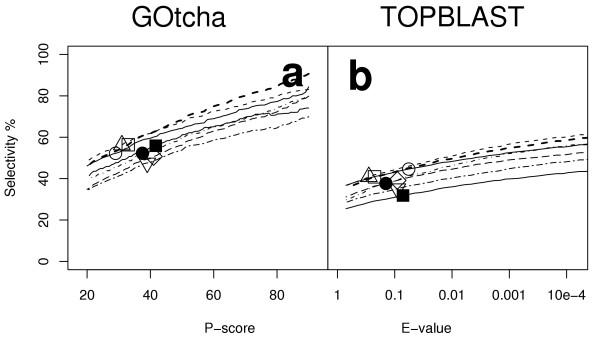
**Selectivity versus cutoff for assignment of GO terms using all evidence codes. **(a) GOtcha with P-score cutoff (a) TOPBLAST with *E*-value cutoff. Key: ○ *Arabidopsis thaliana*; △ *Drosophila melanogaster*; □ *Homo sapiens*; ● *Plasmodium falciparum*; ■ *Vibrio cholerae*; ◇ *Caenorhabditis elegans*; ▽ *Saccharomyces cerevisiae*.

The specificities of function prediction for both GOtcha and TOPBLAST are illustrated in Figure 3. Figure 3a shows the proportion of predictions by GOtcha that are correct with a P-score above the cutoff on the *x*-axis. At a P-score cutoff of 50%, the selectivity of GOtcha is 61.4% (54–68% s.d. 4.9). Figure 3b shows the proportion of predictions by TOPBLAST that are correct with an *E*-value below the cutoff on the *x*-axis. At an *E*-value of 10^-4 ^TOPBLAST shows a selectivity of 53.4% (43–60% s.d. 5.7). Accordingly, GOtcha outperforms TOPBLAST with improved coverage and better selectivity for each genome examined. Both the GOtcha and the TOPBLAST analyses include gene associations that are children of obsolete (GO:0008369) and the three 'unknowns' (cellular_component_unknown, GO:0008372; molecular_function_unknown, GO:0005554; biological_process_unknown, GO:0000004). The obsolete terms comprise a very small proportion (1.5% mean 0 – 3.1% s.d. 1.1) of the total number of annotations (shown in Table 1) and would not be expected to have any significant effect on the results. The three 'unknowns' however are considered to be valid function descriptions. They indicate a clearly observed similarity to a sequence with a function that has not been determined more specifically.

### Function annotation excluding IEA annotations

**Figure 4:**
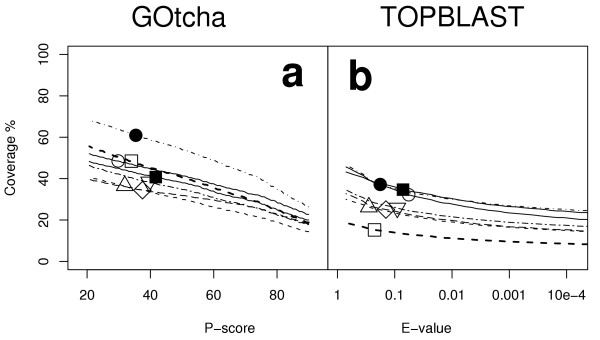
**Coverage vs cutoff for assignment of GO terms excluding IEA evidence codes. **(a) GOtcha (b) top informative BLAST hit. Key: ○ *Arabidopsis thaliana*; △ *Drosophila melanogaster*; □ *Homo sapiens*; ● *Plasmodium falciparum*; ■ *Vibrio cholerae*; ◇ *Caenorhabditis elegans*; ▽ *Saccharomyces cerevisiae*.

Function assignment was repeated using the same BLAST search results but excluding the IEA coded gene-associations. Figure 4 illustrates the recall rate for function assignments. Recovery was lower in all but one genome compared to when IEA terms were included. GOtcha retrieved 39% (30–54 s.d. 7.3) of annotations with a P-score above 50%. This is 83% (54–100 s.d. 14) of the proportion of annotations retrieved by GOtcha when IEA based term associations are included. TOPBLAST retrieved 18% (9–25 s.d. 5.3) of annotations with an *E*-value below 10^-4^. This is 31–81% of the proportion of annotations retrieved when IEA based term associations are included. TOPBLAST only recovers 47% (23–63 s.d. 11) of the number of annotations recovered by GOtcha.

The number of annotations per sequence was reduced by comparison to the data shown in Figure 2 though the trends were very similar (Data not shown). The difference between the analysis with and without IEA terms is consistent with the relative numbers of IEA and non-IEA annotations provided by the genome projects as there are only 9.7% (3.8–14.7 s.d. 4.0) GO term associations per sequence, 62% (23–100% s.d. 30) of the number of GO term associations per sequence when IEA terms are included.

**Figure 5:**
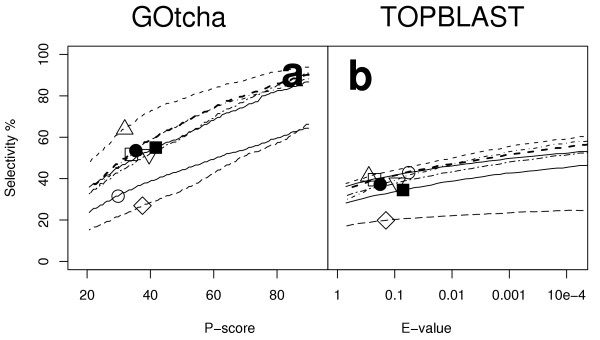
**Selectivity versus cutoff for assignment of GO terms excluding IEA evidence codes. **(a) GOtcha with P-score cutoff (a) TOPBLAST with *E*-value cutoff. Key: ○ *Arabidopsis thaliana*; △ *Drosophila melanogaster*; □ *Homo sapiens*; ● *Plasmodium falciparum*; ■ *Vibrio cholerae*; ◇ *Caenorhabditis elegans*; ▽ *Saccharomyces cerevisiae*.

Figure 5 illustratess the selectivity for the analyses with IEA terms excluded. Figure 5a shows the proportion of assosciations correctly predicted by GOtcha with a P-score above the cutoff on the *x*-axis. Figure 5b shows the proportion of assosciations correctly predicted by TOPBLAST with an *E*-value below the cutoff on the *x*-axis. GOtcha with a P-value cutoff of 50% shows a selectivity of 60% (35–79% s.d. 14). TOPBLAST with an *E*-value of 10^-4 ^shows a selectivity of 49% (25–59% s.d. 11). In all cases except that of *Arabidopsis *GOtcha shows a clear improvement over TOPBLAST with a mean improvement in selectivity of 1.2 fold (0.85 – 1.4 s.d. 0.17).

One issue with excluding IEA annotations is that the coverage of functions in the genome is lowered. This inevitably will lead to a higher number of positives that have incorrectly been assigned as false as a result of the incomplete sequence annotations. Despite excluding terms for which there is no annotation to the ontology under examination, the results are skewed by assigning a proportion of true positives as false positives. This indicates that the method is performing more poorly than is in fact the case. We have examined the nature of the false positives in more detail below.

### A metric for quantitative assessment of function annotation

Comparing function assignment methods is difficult. Typically the standard against which they are assessed is an incompletely annotated dataset. Both a lack of experminental data confirming potential functions and a lack of knowledge about potential functions can lead to the standard data being less perfectly annotated that would be desired. It is not realistically possible in an automated analysis to cope with unrecorded true positives that are registered in the analysis as false positives. It is therefore the case that any analysis of accuracy can only give an estimate of minimum accuracy.

Accuracy can also be difficult to compare between two methods that annotate to different subsets of GO. One method may only annotate to relatively general terms, allowing for a better claimed specificity than a method that attempts to annotate to a more specific level. GOtcha predicts at all levels of the GO hierarchy. It assigns a probability to every combination of GO term – sequence association and should be compared to other function assignment algorithms using a global metric, one which can account for over-specificity and under-specificity in a set of predictions as well as incorrect assignment.

Ouzounis and Karp [4] described the TABS system for qualitative assignment of function annotation to eight categories. The TABS categories are reproduced in Table 2.

When applied to annotation using a DAG such as GO the number of potential categories is reduced from the eight described in TABS to three. TABS was developed to compare annotations where the terms used are not implicitly related through a computable structure such as a DAG. As we are using a DAG where ancestor nodes are implicitly associated with the gene through direct association of a child node, the prediction for a particular sequence becomes a set of GO terms (the nodeset) comprising all nodes that match the prediction rather than just the most specific terms. The accuracy of a prediction can then be assessed by observing the presence of nodes in both the node sets for annotations and for the predictions rather than assigning qualitative values. The more distant a given prediction node set is from the annotation node set, the smaller a proportion of nodes (GO terms) they will have in common.

The effect of a quantitative approach on the TABS categories is as follows: TABS category 0 is unchanged. This is an exact match and is represented by the presence of the term in the node sets for both original annotation and current prediction. TABS category 1 is no longer relevant. A controlled vocabulary is being used so there is no scope for typographical errors of the type described by Ouzounis and Karp or by Tsoka [21] or Iliopoulos [22]. TABS category 2 is also irrelevant. GO has no undefined terms (though a small proportion of terms lack complete descriptions) and all annotation sources are attributed using evidence codes and references. TABS category 4 is an extreme case of category 3. Both these categories are represented by the existence of a function annotation in the original annotation node set but not in the predicted node set. Likewise TABS category 7 is an extreme case of TABS category 6. In many cases a false positive is represented as an underprediction in the true branch of the GO DAG and an overprediction in a false branch. TABS category 5 describes the mechanism of occurrence of an error rather than the error itself and is not relevant to this analysis. In this analysis we reduce the eight TABS categories describing the accuracy of a function prediction for an individual sequence to three categorise that describe each node in the nodeset comprising a function prediction for an individual sequence. These categories correspond to false positive, false negative and true positive nodes. A particular sequence annotation node set could potentially contain nodes from all three categories.

#### Quantitation of the analysis

**Figure 6:**
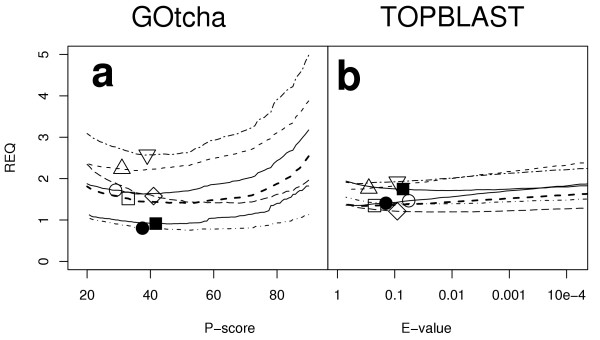
**Relative Error Quotient (*REQ*) vs cutoff for assignment of GO terms. ***REQ *is defined in the text. (a). GOtcha analysis. (b). Top informative BLAST hit analysis. Key: ○ *Arabidopsis thaliana*; △ *Drosophila melanogaster*; □ *Homo sapiens*; ● *Plasmodium falciparum*; ■ *Vibrio cholerae*; ◇ *Caenorhabditis elegans*; ▽ *Saccharomyces cerevisiae*.

Given two sets A and B corresponding to a given annotation set and a predicted set (each node in the set comprising a sequence – GO term association) we are interested in the true matches (intersection of A and B, *n *∈ *A *∩ *B*), false positives (term associations in B but not in A, *n *∈ *B*, *n *∉ *A*) and the false negatives (term associations in A but not in B, *n *∈ *A*, *n *∉ *B*). The aim of any prediction method is to maximise the number of matches (true positives) whilst minimising the errors (false positives and false negatives). The number of true negatives does not need to be considered as this number is very large and essentially constant over the analysis. We can use the following relation as an error quotient to assess prediction methods.


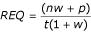


where *REQ *is the Relative Error Quotient, *n *is the total false negatives, *p *is the total false positives, *w *is a weighting factor and *t *is the total true positives. A low *REQ *represents a low proportion of errors. A higher *REQ *indicates a higher proportion of errors. Such a measure is dependent upon the population of the node set which in turn is dependent upon the cut off used for selecting predictions in the node set. Figure 6a shows the change in *REQ *with respect to P-score cutoff for the GOtcha analysis and Figure 6b the *REQ *with respect to *E*-value cutoff for the TOPBLAST assignments. A weighting factor of 1 was used in both cases, thus giving equal weight to both false positives and false negatives. In this figure the minima indicate optimum cutoffs for maximising the similarity between annotation and prediction nodesets. The GOtcha results (Figure 6a) indicate broad minima, suggesting that small differences in cut off selection may have only a slight effect on the accuracy of the results. The minima for BLAST are difficult to see as they are skewed to very high *E*-values as a result of a large proportion of false negatives. This indicates that the TOPBLAST search is rejecting important information present in matches with *E*-values approaching 1, much higher than those normally used for genome annotation. The REQ metric therefore appears to perform quite robustly. This metric assigns identical weight to each GO term association. More complex weighted measures of semantic similarity have been proposed by Lord and coworkers for searching databases based on annotation [23] but these are difficult to apply to the present problem in a manner that uses a non-arbitrary weighting.

**Figure 7:**
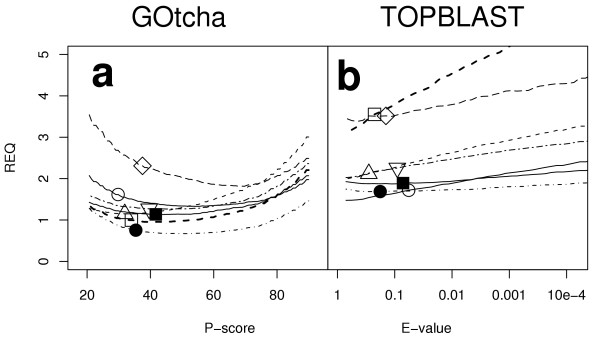
**Relative Error Quotient (*REQ*) vs cutoff for assignment of GO terms. ***REQ *is defined in the text. IEA terms were excluded from this analysis. (a). GOtcha analysis. (b). Top informative BLAST hit analysis. Key: ○ *Arabidopsis thaliana*; △ *Drosophila melanogaster*; □ *Homo sapiens*; ● *Plasmodium falciparum*; ■ *Vibrio cholerae*; ◇ *Caenorhabditis elegans*; ▽ *Saccharomyces cerevisiae*.

In the absence of IEA annotations the spread of the REQ curves changes dramatically as shown in Figure 7. Figure 7a illustrates the REQ for GOtcha with the differences between the genomes far less marked than for Figure 6a. In contrast, the REQ for TOPBLAST is shown in Figure 7b and shows much higher and more diverse REQ than when IEA terms are included (Figure 6b).

Minimum *REQ *(i.e. maximum accuracy) has been determined for both GOtcha and top BLAST hit annotation sets, both with and without the use of automated annotations (IEA evidence code) for transitive function assignment (Table 3). When automated annotations (IEA codes) are included in the analysis, there is no significant difference between the minimum *REQ *obtained using GOtcha or that from TOPBLAST. The minimum REQ for TOPBLAST is obtained at very high *E*-values, 0.011–0.71 when IEA terms are included (Figure 6) and 0.12–0.71 when IEA terms are excluded (Figure 7). When IEA annotations are excluded from the analysis GOtcha performs significantly better than TOPBLAST (p ≤ 0.016 using the Wilcoxon signed rank test). GOtcha excluding IEA terms performs better (though this small number of genomes does not give a statistically significant result) than when IEA annotations are included (mean change: 15% reduction in *REQ *s.d. 11%, p = 0.2 using the Wilcoxon signed rank test). It may be that the annotation set used as the reference in comparing these results was incomplete. This would result in some true positives being incorrectly assigned as false positives with a corresponding increase in *REQ*. However, this would apply similarly to GOtcha and to the top BLAST hit analysis.

### Assessment of incorrectly assigned false positives

Samples of the false positive function predictions by GOtcha with the highest P-scores from three *P. falciparum *chromosomes (representing the three genome centres in the Malaria Genome Sequencing Consortium) were assessed by hand to give an indication of the completeness of the curated annotations. Results for selected sequences in this set are shown in additional file 1. Twenty sequences were examined: ten from chromosome 12, and five taken from each of chromosomes 2 and 3. Chromosome 3 was the first to be sequenced and is the most carefully annotated of the chromosomes. In each case the sequences selected were those with the highest scoring false positive function assignments. Representative results from the analysis of GOtcha annotation with and without IEA terms are available as supplementary material. The proportion of correct annotations generally performed better than the P-score would suggest. Taking a P-score of > 50% as a cutoff, most GOtcha predictions agreed with the function assigned by the curator. The false positives fell into several categories:

#### Differences in curator judgement

In some examples, genes that were annotated as encoding hypothetical proteins could be re-annotated based on GOtcha predictions. GO terms had not been assigned during the manual curation phase of the *P. falciparum *genome project if no function had been identified during the first-pass automatic annotation. However, the addition of GO terms to sequences by GOtcha prompted the original annotation to be re-evaluated. For example, PFL1875w shows a hit to the Pfam K+ tetramerisation domain (Pfam:0224, E = 10^-9^) supports the GOtcha annotation although it is at a level that genome annotators may feel is marginal. In PFL1780w, stronger supporting evidence (a hit with E = 10^-12 ^to Pfam:04140, isoprenylcysteine carboxyl methyl transferase domain) indicates again that GOtcha can suggest GO annotations that have been previously overlooked.

In several examples, GOtcha predicted either additional functions or more specific GO terms to describe previously annotated functions. PFC0495w encodes a putative aspartyl protease. When all evidence codes were included, a molecular function of pepsin A activity is predicted. This protein matches pepsin A domains defined by the InterPro entry IPR001461 ('Peptidase_A1 pepsin A'), thus the term from GOtcha is likely to be correct.

#### Human error

PFL2465 encodes a thymidylate kinase, which was correctly annotated by GOtcha as being involved in dTTP biosynthesis. GOtcha also indicates 'dTDP biosynthesis' as a suitable GO process term. Thymidylate kinase catalyses the synthesis of dTDP, a necessary step in dTTP biosynthesis. However, the human annotator missed the fact that dTDP biosynthesis is not a 'part of' dTTP biosynthesis within the ontology structure and in such cases, terms describing both processes must be employed.

Sometimes, GOtcha highlighted erroneous omissions in the GO annotation of the *P. falciparum *genome, many of which have arisen from retrospective corrections and amendments to gene models. For instance, GOtcha provides detailed annotation for a putative ATPase synthase F1 alpha subunit (PFB0795w) almost completely lacking useful GO terms. GOtcha also suggested GO terms relating to translation elongation for PFL1710c. A highly significant hit to Pfam:00009 (Elongation factor Tu GTP binding domain, E = 10^-46^) indicates that this GOtcha prediction may well be more accurate then the original genome annotation.

#### IEA vs non IEA

Annotations performed with IEA terms appeared to be more specific than those where IEA terms were excluded. In many cases, such as PFC0495w, the difference was quite pronounced. Here the protein was implicated in 'proteolysis and peptidolysis' when all annotations were included but filtering out IEA annotations resulted in the more general, and less useful, description of 'metabolism'.

#### Real false positives

Out of the 20 genes inspected, PFL1825w was the only example where GO terms were incorrectly suggested for the biological process, molecular function and cellular component aspects of GO. In other cases, mis-annotations often had low I scores (predictions made with P-scores > 50% but very low associated I-scores ≪ 0.1) or were due to terms taken from slightly too far down a branch in the ontology structure. For example 'ATP-binding and phosphorylation-dependent chloride channel' was predicted for PFB0795w, an ATP synthase.

The cellular component of gene products are hard to annotate – often BLAST is insufficient to recognise the targeting information encoded in signal and transit peptides and specific signal sequence detection methods such as PSORT II [24] must be used instead. GOtcha consequently made incorrect predictions of subcellular localisation in some cases. For instance PFL1710c is annotated as having mitochondrial and apicoplast localisation based on separate lines of evidence [15] but GOtcha predicted cytoplasmic localisation with a P-score of 52%.

It is hard to measure what proportion of the calculated false positives does in fact represent serious mis-annotation. Although the hand analysis may provide representative examples, it is too small to be of statistical significance. Genuine false positives (with high P- and I-scores) were fewer than would be expected from the P-score. Despite the small sample size, these results show that GOtcha performs well as a guide to the manual assignment of GO terms. Not only can it provide suggestions for more granular annotation but it can highlight terms that would otherwise be missed by a human annotator.

## Discussion

### Data interdependency and annotation accuracy

One of the major problems facing assessment of function assignment is the separation of annotation and test datasets. In this analysis we have tackled this issue by taking individual genome datasets as the test sets and using other genome datasets for the annotation source from which to transitively assign function. The scoring mechanism used for estimating accuracy values is independent of both test and annotation datasets, since it makes use of sequences that are found in neither. Whilst the sequences are independent, the annotations associated with these sequences may not be. Many of the computationally assigned annotations are derived from analyses involving the 'independent' datasets and can therefore not be regarded as entirely independent. IEA annotations are primarily obtained from sequence similarity searches. As a consequence it is not surprising that the results obtained for both GOtcha and TOPBLAST when IEA annotations are included are so similar. Interestingly, when IEA based annotations are excluded from the TOPBLAST analysis, the *REQ *goes up. This may well indicate a degree of inaccuracy in the IEA based annotations, or incomplete coverage by the human curated annotations. GOtcha, however, makes a significantly better use of the BLAST search result in the quality and coverage of the annotation.

#### False positive/false negative balance in the relative error quotient

**Figure 8:**
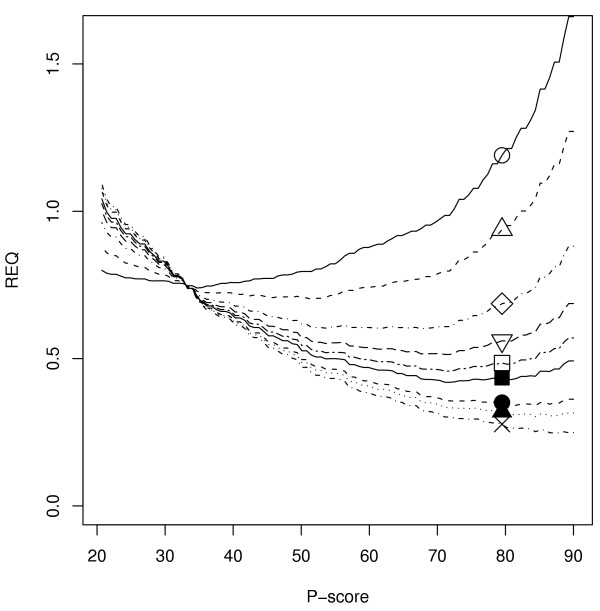
**The effect of different weights on REQ. **The REQ for GOtcha predictions of GO term associations for the human proteome was calculated with weighting factors of 0.5 (open circle), 1, 2,3,4, 5, 7, 10 and 15 (cross).

The REQ analyses performed weighted under prediction errors (false negatives) equally to over prediction errors (false positives). In order to examine the effect of the weighting on *REQ*, the GOtcha predictions for the human genome were compared to the genome consortium annotations with weights ranging from 0.5 to 15 (Figure 8). As expected, an increased emphasis on false positives shifts the minimum *REQ *towards a higher P-score cutoff. Weighting can be adjusted depending on the aims of the study in question. The minimum *REQ *should give the best tradeoff between accuracy and coverage and can be used to estimate an optimum P-score cutoff for transitive assignment of function. Investigations that emphasise accuracy over coverage may increase the weight to reduce false positives. Investigations with less concern for accuracy but a greater emphasis on coverage will use a lower weight for minimal *REQ *determination to increase coverage.

The metric presented here is an objective measure of method performance but has some drawbacks. Using the *REQ *as described in this paper, each term in the nodeset is weighted equally. This may not be the most appropriate measure. The granularity of terms in Gene Ontology is not constant across the ontologies, nor is it readily quantifiable. This may lead to bias in the metric, where differences in the presence or absence of closely related terms is weighted equally to presence or absence of more distantly related terms even though they have the same graph path distance between them. There is also the issue of prevalence. Some terms occur in almost every nodeset, others are less prevalent. The most appropriate form for a quantitative metric will need to be examined in future work.

Transitive function assignment is limited by the sensitivity of the underlying search method and the scope of the dataset being searched. The GOtcha method of preparing a weighted composite view of the functions from a complete set of search results provides a significant improvement in the annotation of sequences when compared to a method that selects the most significant annotated hit. GOtcha also provides a confidence measure for the putative function assignments, allowing for the determination of an appropriate level of specificity for the annotation set. Hennig and co-workers examined the ability of BLAST analysis to transitively assign function from distant taxa, concluding that for the majority of cases, GO-based annotation would give a good result [14]. In this study we have performed seven-fold cross validation with seven distinct genomes across the taxonomic range. It is intended to improve the performance of this method by including further genomes and updating the annotations on those already used.

## Conclusions

The GOtcha method has several significant advantages over the transitive assignment of function by TOPBLAST. Firstly each function assignment has a directly understandable accuracy estimate that can be interpreted without any knowledge of the prediction methodology. This accuracy estimate is function-specific, unlike general rules of thumb that are applied to interpretation of BLAST search results. Secondly, the GOtcha method provides much greater coverage than a top annotated match approach, annotating more sequences with reasonable confidence. In many cases it provides annotations for sequences that otherwise would have no annotations. Finally, it provides term specific annotation accuracy estimates. This is a significant advantage over TOPBLAST where every term in the set predicted for an individual sequence has the same value and a biologist interpreting the results is given little indication of which terms can reasonably be accepted. In contrast, GOtcha provides individual P-scores for each term. This allows a rapid visual examination of the prediction as a graph or a list, indicating appropriate points at which experimental verification may best be directed.

In order to assess the accuracy of annotations to tree-like ontologies we have developed an objective flexible scoring metric that provides a global analysis, including assessment of both false positives and false negatives. This metric also provides a means for comparison of methods that is not dependent on the selection of any particular parameter threshold or cutoff in the scoring method used.

The underlying mapping methodology applied in GOtcha can readily incorporate other search methods that provide a more sensitive similarity search. Combining search methods should also provide a better coverage of sequence space occupied by distant homologues [25], and such potential improvements are the subject of further work.

## Methods

### Data sources

All data were obtained in the same week (week 9, 2003) to provide a consistent time point at which to perform the analysis.

#### Sequence data

Malaria (*Plasmodium falciparum*) sequence data for the recently determined genomic sequence [15] were obtained from the malaria consortium. The whole genome annotated peptide set was dated 3 October 2002 and comprised 5334 peptides varying in length from 17 to 10589 amino acid residues.

Fruit fly (*Drosophila melanogaster*) data were obtained from Flybase [26] as release 3.1 of the annotated full genome transcript set. This set contained 18484 transcript sequences corresponding to 13656 genes. A non-redundant set was created for subsequent analysis by selecting the longest transcript to represent each gene. Transcript lengths varied from 15 to 69162 nucleotides (5 to 23054 amino acid residues).

Yeast (*Sacchyromyces cerevisiae*) data were obtained from the Sacchyromyces Genome Database [27]. The set of translated open reading frames for the whole genome was used and comprised 6356 peptides varying in length from 25 to 4911 amino acid residues.

Cholera *Vibrio cholerae *data were obtained from The Institute for Genomic Research [28]. The dataset contained 3836 sequences varying in length from 26 to 4588 amino acid residues.

Human (*Homo sapiens*) data were obtained from Swiss-Prot using the conceptual complete human proteome from the Swiss-Prot/EnsEMBL collaboration dated 6 March 2003 [29]. The dataset contained 39080 proteins with lengths varying from 3 to 34350 amino acid residues.

Worm (*Caenorhabditis elegans*) data were obtained from Wormbase release 97 [30]. The dataset contained 30753 peptides varying in length from 4 to 13100 amino acid residues.

Thale cress (*Arabidopsis thaliana*) data were obtained from The Arabidopsis Information Resource [31]. The complete genome peptide set dated 31 July 2002 was used. The dataset contained 27288 sequences varying in length from 20 to 4707 amino acid residues.

Each data set was formatted for BLAST searching with the formatdb program from the BLAST2 suite [32,33]. The URI for each genome dataset are listed in Table 4

#### Gene association and Gene Ontology data

Data for the Gene Ontology and gene associations for all proteome sets except Arabidopsis were downloaded from the Gene Ontology CVS repository in week 9, March 2003, parsed and loaded into a relational database. Arabidopsis data were obtained from The Arabidopsis Information Resource using gene association data dated 13 February 2003. A flat file database containing the Gene Ontology and gene association data was developed and indexed to allow rapid retrieval of individual entries by custom written Perl modules. The number of annotated sequences in each data set is shown in Table 1.

#### Software

The BLAST2 programs were obtained from NCBI. Analyses were performed on a cluster of 50 HP Netserver L1000 dual processor machines configured with two 1.4 GHz Pentium III processors, 70 Gb hard disk, 2 Gb RAM and running a customised Linux operating system. Job scheduling was performed with Grid Engine (Sun Microsystems). Results were stored in a relational database (PostgreSQL version 7.3) or as flat files where appropriate. BLAST result parsing was performed with the BioPerl toolkit (release 0.7) [34]. Sequence manipulation was performed with EMBOSS [35]. All processing scripts were written in Perl. A set of Perl modules were developed for accessing and manipulation of data entries.

## Methods

### GOtcha method overview

**Figure 9:**
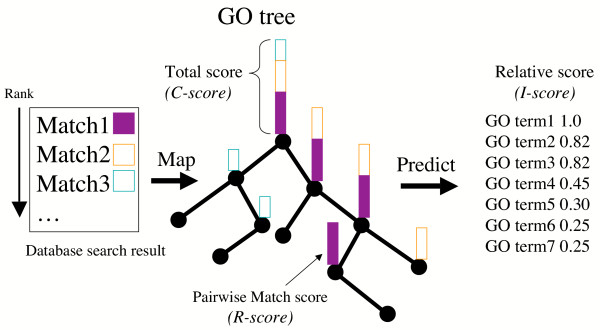
**The GOtcha method. **1. A query sequence is subjected to a database search. The search results are processed to give a list of pairwise matches with associated R-scores. 2. The R-score for the pairwise match is added to the total score for each GO term associated with that match sequence. 3. The C-score is calculated as the natural logarithm of the total score at the root node. The I-score for each node is calculated as the ratio of the total node score to the root node.

The GOtcha method is illustrated by a cartoon in Figure 9. We have implemented this method by searching against a cohort of seven well defined and annotated genomes. To predict the association of GO terms with a specific individual gene product a BLAST search is run against each genome data set using the appropriate program (blastx/tblastn when *D. melanogaster *was the query/subject set, blastp otherwise). Default parameters were used (Maximum expectancy score 10; maximum list sizes 250 and 500 hits). Each sequence database search produces a ranked set of sequences similar to the query sequence. The search result for each genome database search is parsed and a list of pairwise matches between the query sequence and the subject database sequences obtained.

For each similarity match between the query sequence and a database sequence, a set of GO terms corresponding to the gene-associations for the database sequence is retrieved from the appropriate gene-association dataset. The set of GO terms and all ancestral terms (the nodeset) are assigned a score R = max { -log_10_(*E*), 0 } where *E *is the expectancy score for that pairwise match. In this way the whole subtree to the root node is assigned the R-score. The GOtcha method allows mappings obtained from many sequence matches to be combined. For each node (which corresponds to an individual GO term, either directly associated or the ancestor of an associated GO term), R-scores for all pairwise matches which contain annotation to that node are summed and normalised to the total R-score for the root node of that ontology (Cellular Component, GO:0005575; Molecular Function, GO:0003674; or Biological Process, GO:0008150). This normalisation gives an internal relative score (the I-score), producing a weighted composite subgraph of the GO. This normalisation effectively removes bias in the E-value due to database size or search program used. A confidence measure is calculated as loge of the root node score (the C-score). Accordingly, this provides two measures for an individual predicted gene-association; A score relative to the other predicted gene-associations in the node set (the I-score) and a score for the function prediction as a whole (the C-score).

Each genome was searched individually and I-score and C-score for each GO term association were averaged across all genome searches that provide at least one annotated pairwise match. Averaging across genomes in this way provides some correction for individual genes with exceptionally high copy numbers in certain genomes. In this paper the term 'function prediction' relating to an individual sequence refers to a prediction of a set of GO term – sequence associations (also referred to as a node set). Averaging of the individual search results avoids the over-representation of large genomes in the final annotation set and allows the final result to be weighted towards a particular taxonomic grouping should that be desired. Each gene association represents a function assignment of a gene product with a GO term and is annotated with an evidence code providing an indication of the reliability of a particular annotation. The GOtcha method allows specific classes of annotation, such as those derived exclusively from computational analyses, to be excluded from the analysis if required.

### Background accuracy estimates for individual GO terms – P-score table construction

Although higher C-score and I-score values correspond to greater confidence in the transitive assignment of function than lower C-score or I-score values, it is not immediately apparent how these values should be interpreted. Examination of preliminary results indicated that there was considerable variation between GO terms in the confidence that can be placed in a prediction with a given I-score and C-score (data not shown). Accordingly we have created an empirically based estimate of accuracy (the P-score, expressed as a percentage) that can be used to indicate confidence in the prediction of association between a GO term and a gene product.

A background set of 518226 annotated sequences from the SwissProt gene associations were included in the accuracy estimate after excluding taxa corresponding to the search databases and their subspecies. All background sequences were subject to a search against all 7 species specific datasets and a set of function predictions obtained as described above. A scoring table for each GO term was prepared by segregating all predictions for that GO term on I-score and C-score. I-scores were divided into ten rows by dividing the range (0 – 1) evenly. C-scores were divided into columns by unit ranges (0–1, 1–2, 2–3 and so on). This gave rise to approximately one hundred cells for each GO term table. Each prediction was assigned to a cell based upon its I-score and C-score. The overall accuracy of each cell was determined by comparison of the predicted associations in that cell to the annotations provided by the GO Annotation project (GOA) and calculated as the proportion of true positives to the sum of true and false positives. The table for a specific GO term was then used to deliver the P-score based on any given I-score and C-score pair for a predicted association between that GO term and the query sequence. A similar set of tables was constructed from background analyses from which terms with IEA associations were excluded. For GO terms where there are few datapoints with which to estimate accuracy reliably, accuracy estimation falls back to a scoring table that combines results over all GO terms from that ontology with the same number of ancestors.

### Function assignment by top informative BLAST hit

The same BLAST searches used for function assignment with the GOtcha method were analysed. Function assignments for the nodeset corresponding to the top annotated BLAST match (TOPBLAST) for each genomic dataset were transferred to the query sequence with a score corresponding to the *E*-value for that hit.

## List of abbreviations

DAG, Directed Acyclic Graph. URI, Uniform Resource Identifier. BLAST, Basic Local Alignment Search Tool. TOPBLAST, Top annotated BLAST match. Perl, Practical Extraction and Report Language. GO, Gene Ontology. TABS, Transitive Annotation Based Score. NCBI, National Centre for Biological Information. s.d., Standard deviation.

## Authors' contributions

The GOtcha method was devised and implemented by DMAM who also prepared the manuscript. MB performed the manual assessment of false positives and provided feedback on the presentation of results. GJB provided essential guidance for the performance assessment and revision of the manuscript.

## Supplementary Material

Additional File 1 The supplementary data contains representative examples from the manual assessment of false positives. It is portrayed in tabular format and indicates the benchmark annotation, the highest scoring predicted incorrect annotation by GOtcha and the lowest scoring predicted annotation by GOtcha. Click here for file

## Figures and Tables

**Table 1:** Sequences and annotations for each dataset.

			Sequences annotated by ontology		
Dataset	Total associations	Total sequences	Cellular Component	Molecular Function	Biological Process

*Arabidopsis thaliana*	290952(94824)	20108(7969) [451]	14851(2115)	14467(7555)	10454(3481)
*Drosophila melanogaster*	129694(29311)	7536(7536) [0]	3613(3589)	6528(6520)	3730(3723)
*Homo sapiens*	409153(67357)	21251(9074) [659]	13723(6516)	19362(7328)	17080(7707)
*Plasmodium falciparum*	36952(32536)	2406(2209) [41]	2061(1227)	2094(2094)	2044(2044)
*Saccharomyces cerevisiae*	136938(36267)	6910(6849) [0]	6751(6751)	6831(6831)	6899(6838)
*Vibrio cholerae*	42616(42616)	2924(2924) [27]	189(189)	2721(2721)	2923(2923)
*Caenorhabditis elegans*	109360(18626)	6916(1870) [199]	3054(650)	5746(282)	5102(1557)

Values in parentheses do not include IEA associations. Values in [brackets] are sequences with annotations that are children of obsolete (GO:0008369).

**Table 2:** TABS scheme for qualitative assessment of annotation accuracy.

Category	Description	Comment
0	Total agreement	Original annotation is correct, but annotations may be only semantically (but not computationally) identical
1	Typographical error	Original annotation contains typographical errors that may be propagated in the database
2	Undefined source	Original annotation contains undefined terms, non-homology based predictions, and so on
3	Under-prediction	Original annotation predicts a nonspecific biochemical function although a more detailed prediction could have been made
4	False negative	Original annotation does not provide predicted function although there is sufficient evidence to characterize the query protein
5	Domain error	Original annotation overlooks different domain structure of query and reference proteins
6	Over-prediction	Original annotation predicts a specific biochemical function without sufficient supporting evidence
7	False positive	Original annotation predicts function without any supporting evidence

(from Ouzounis and Karp, Genome Biol. 2002;3(2):COMMENT2001)

**Table 3:** Minimum REQ values for seven datasets using two methods for annotation.

Dataset	GOtcha		Top Hit	GOtcha (-IEA)		Top Hit (-IEA)		
	
	*REQ*	cutoff	REQ	cutoff	REQ	cutoff	REQ	cutoff
*Arabidopsis thaliana*	1.63	36	1.35	0.56	1.33	54	1.48	0.71
*Caenorhabditis elegans*	1.41	68	1.19	0.023	1.82	70	3.38	0.45
*Drosophila melanogaster*	2.20	35	1.74	0.71	1.15	31	1.99	0.71
*Homo sapiens*	1.40	52	1.34	0.35	0.95	39	3.12	0.71
*Plasmodium falciparum*	0.75	51	1.39	0.023	0.67	51	1.69	0.28
*Saccharomyces cerevisiae*	2.56	38	1.88	0.41	1.27	45	2.03	0.71
*Vibrio cholerae*	0.91	45	1.71	0.011	1.13	43	1.87	0.11
Mean (+/- SD)	1.55 (0.60)		1.51 (0.24)		1.19 (0.32)		2.22 (0.67)	

Cut off values for GOtcha are P-score. Cut off values for BLAST are *E*-value.

**Table 4:** Genome Project URIs for datasets used in the study.

Dataset	Genome Project Site URL
Malaria (*Plasmodium falciparum*)	
Fruit fly (*Drosophila melanogaster*)	
Yeast (*Sacchyromyces cerevisiae*)	
*Vibrio cholerae*	
Human (*Homo sapiens*)	
Worm (*Caenorhabditis elegans*)	
Thale cress (*Arabidopsis thaliana*)	ftp://tairpub:tairpub@ftp.arabidopsis.org
