# Supplementary material for: GOtcha: a new method for prediction of protein function assessed by the annotation of seven genomes
Source: BMC Bioinformatics. 2004 Nov 18;5:178. doi: 10.1186/1471-2105-5-178 (PMC535938; doi:10.1186/1471-2105-5-178)
Supplement: Additional File 1 — The supplementary data contains representative examples from the manual assessment of false positives. It is portrayed in tabular format and indicates the benchmark annotation, the highest scoring predicted incorrect annotation by GOtcha and the lowest scoring predicted annotation by GOtcha. [file 1471-2105-5-178-S1.pdf]

Supplementary information

Each column lists the most granular correct terms with P-score > 50%. More than one term indicates hits to more than one branch. Each term is listed as GO ID, I-score ± s.d., P-score, (description)

Manual assessment of false positives – IEA annotation included

**Gene name and annotated product description**	**Original human GO annotation**	**With IEA annotations**	
		Lowest scoring true positive	Highest scoring false positive with P-score > 50%
PFL2465 Thymidylate kinase	GO:0006235 (dTTP biosynthesis)	GO:0006235, 0.59±0.48, 98% (dTTP biosynthesis)<br>GO:0006233, 0.59±0.48, 100% (dTDP biosynthesis)	n/a
	GO:0008372 (cellular_component unknown)	n/a	n/a
	GO:0004798 (thymidylate kinase activity)<br>GO:0005524 (ATP binding)	GO:0004798, 0.9893±0.0142, 100% (thymidylate kinase activity)<br>GO:0005524, 0.40±0.49, 68% (ATP binding)	n/a
PFL1875w hypothetical protein	GO:0000004 (biological process unknown)	GO:0008152, 0.53±0.38, 67% (metabolism)<br>GO:0006813, 0.65±0.46, 100% (potassium ion transport)	n/a
	GO:00016020 (membrane)	GO:0008076, 0.66+/-0.47, 69% (voltage-gated potassium channel complex)	n/a
	GO:00005554 (Molecular function unknown	GO:0005249, 0.65+/-0.46, 58% (voltage-gated potassium channel)	n/a
PFL1835w hypothetical protein	GO:0000004 (biological process unknown)	n/a	GO:0009190, 0.0009±0.0019, 88% (cyclic nucleotide biosynthesis)<br>GO:0006304, 0.0015±0.0030, 56% (DNA modification)
	GO:00016020 (membrane)<br>GO:0020011 (apicoplast)	GO:0016021, 0.0069±0.0124, 74% (integral to membrane)	GO:0005891, 0.01±.0.01, 74% (voltage-gated calcium channel complex)<br>GO:0005884, 3e-05±7e-05, 56% (actin filament)
	GO:00005554 (Molecular function unknown	n/a	GO:0015078, 0.001±0.003, 96% (hydrogen ion transporter)

			GO:0004601, 0.0003±0.0007, 68% (peroxidase)<br>GO:0003890, 3e-05±7e-05, 96% (beta DNA polymerase)<br>GO:0003889, 0.0001±0.0002, 100% (alpha DNA polymerase)<br>GO:0003902, 0.0002±0.0005, 56% (DNA-directed RNA polymerase III)<br>GO:0015399, 0.0758±0.1380, 58% (primary active transporter)<br>GO:0004842, 0.0196±0.0167, 79% (ubiquitin-protein ligase)<br>GO:0004222, 0.0033±0.0064, 80% (metalloendopeptidase)<br>GO:0005525, 0.0014±0.0021, 64% (GTP binding)<br>GO:0008270, 0.0014±0.0029, 56% (zinc ion binding)
PFL1825w hypothetical protein	GO:0000004 (biological process unknown)	n/a	GO:0015980, 0.8536±0, 65% (energy derivation by oxidation of organic compounds)
	GO:00016020 (membrane)	GO:0005623, 1±0, 68% (cell)	
	GO:00005554 (Molecular function unknown)	n/a	GO:0004026, 0.8536, ±0, 60% (alcohol O-acetyltransferase)
PFL1780w hypothetical protein	GO:0000004 (biological process unknown)	n/a	n/a
	GO:00016020 (membrane)<br>GO:0020011 (apicoplast)	GO:0016020, 0.6512±0.4609, 91% (membrane)	n/a
	GO:00005554 (Molecular function unknown)	GO:0004671, 0.6512±0.4609, 70% (protein-S-isoprenylcysteine O-methyltransferase)	n/a
PFL1710c tetQ family GTPase, putative	GO:0000004 (biological process unknown)	GO:0006414, 0.7538±0.1860, 99% (translational elongation)	n/a
	GO:0005739 (mitochondrion)		GO:0005737, 0.5442±0.4546, 52%

	GO:0020011 (apicoplast)		(cytoplasm)
	GO:0005525 (GTP binding)	GO:0005525, 0.3693±0.3237, 98% (GTP binding)<br>GO:0003746, 0.71±0.17, 99% (translation elongation factor)	
PFC0495w aspartyl protease, putative	GO:0006508 (proteolysis and peptidolysis)	GO:0006508, 0.5244±0.4754, 76% (proteolysis and peptidolysis)	n/a
	GO:0008372 (cellular_component unknown)	n/a	n/a
	GO:0004190 (aspartic-type endopeptidase activity)	GO:0004194, 0.4976±0.4976, 100% (pepsin A)	n/a
PFB0795w ATP synthase F1, alpha subunit, putative	GO:0000004 (biological process unknown)	GO:0015986, 0.3333±0.4082, 100%<br>(ATP synthesis coupled proton transport)	n/a
	GO:0005739 (mitochondrion)	GO:0016469, 0.3437±0.4302, 90%<br>(hydrogen-transporting two-sector ATPase complex)	n/a
	GO:00005554 (Molecular function unknown)	GO:0003936, 0.9638±0.0648, 100%<br>(hydrogen-transporting two-sector ATPase)	GO:0005224, 0.1695±0.3199, 100% (ATP-binding and phosphorylation-dependent chloride channel)

Manual assessment of false positives – IEA annotation excluded.

**Gene name and annotated product description**	**Original human GO annotation**	Without IEA annotations	
		Lowest scoring true positive	Highest scoring false positive with P-score > 50%
PFL2465 Thymidylate kinase	GO:0006235 (dTTP biosynthesis)	GO:0006233, 0.32±0.45, 26% (dTDP biosynthesis)<br>GO:0006235, 0.32±0.45, 26% (dTTP biosynthesis)	GO:0019222, 0.002±00.003, 64% (regulation of metabolism)
	GO:0008372 (cellular_component unknown)	n/a	GO:0005737, 0.66±0.46, 76% (cytoplasm)
	GO:0004798 (thymidylate kinase activity)<br>GO:0005524 (ATP binding)	GO:0004798, 0.98±0.02, 86% (thymidylate kinase activity)	n/a
PFL1875w	GO:0000004 (biological process	GO:0008152,0.85±0.15, 73% (metabolism)	n/a

hypothetical protein	unknown)		
	GO:00016020 (membrane)	GO:0005623, 0.85±0.15, (cell)	GO:0005622, 0.85±0.15, 71% (intracellular)
	GO:00005554 (Molecular function unknown	n/a	GO:0005554, 0.15±0.15, 70% (molecular_function unknown)
PFL1835w hypothetical protein	GO:0000004 (biological process unknown)	GO:0007582, 0.6145±0.2521, 75% (physiological processes) GO:0009987, 0.6223±0.2971, 82% (cellular process)	GO:0008151, 0.5920±0.2932, 70% (cell growth and/or maintenance)
	GO:00016020 (membrane) GO:0020011 (apicoplast)	GO:0016021, 0.0069±0.0124, 58% (integral to membrane)	GO:0005625, 0.0017±0.0035, 65% (soluble fraction) GO:0005624, 0.0001±0.0003, 52% (membrane fraction)
	GO:00005554 (Molecular function unknown)	n/a	GO:0005525, 0.0011±0.0022, 52% (GTP binding) GO:0004930, 0.0011±0.0023, 71% (G-protein coupled receptor)
PFL1825w hypothetical protein	GO:0000004 (biological process unknown)	n/a	GO:0006113, 0.8536±0, 54% (fermentation) GO:0008151,1±0,100% (cell growth and/or maintenance) GO:0006091, 0.8536±0, 82% (energy pathways) GO:0015980, 0.8536±0, 81% (energy derivation by oxidation of organic compounds)
	GO:00016020 (membrane)	GO:0000267, 0.8536±0, 63% (cell fraction)	
	GO:00005554 (Molecular function unknown	n/a	GO:0008374, 0.8536±0, 70% (O-acyltransferase) GO:0016407, 0.8536±0, 70% (acetyltransferase)
PFL1780w hypothetical protein	GO:0000004 (biological process unknown)	GO:0006464, 0.4769±0.4769, 83% (protein modification)	GO:0007323, 0.4769±0.4769, 83% (peptide pheromone maturation)
	GO:00016020 (membrane) GO:0020011 (apicoplast)	GO:0005783, 0.4769±0.4769, 52% (endoplasmic reticulum) GO:0012505, 0.4769±0.4769, 51% (endomembrane system)	n/a
	GO:00005554 (Molecular function unknown	GO:0008171, 0.9769±0.0230, 92% (O-methyltransferase) GO:0008757, 0.9769±0.0230, 92% (S-adenosylmethionine-dependent methyltransferase) GO:0008276, 0.9769±0.0230, 92% (protein	n/a

		methyltransferase)	
PFL1710c<br>tetQ family GTPase, putative	GO:0000004 (biological process unknown)	GO:0006412, 0.4782±0.4060, 56%<br>(protein biosynthesis)	n/a
	GO:0005739 (mitochondrion)<br>GO:0020011 (apicoplast)	n/a	GO:0005737, 0.3462±0.4325, 52%<br>(cytoplasm)
	GO:0005525 (GTP binding)	GO:0005525, 0.1341±0.0840, 54% (GTP binding)<br>GO:0003746, 0.4905±0.2708, 63% (translation elongation factor)	GO:0004930, 0.0017±0.0035, 71% (G-protein coupled receptor)
PFC0495w<br>aspartyl protease, putative	GO:0006508 (proteolysis and peptidolysis)	GO:0008152, 0.6529±0.4620, 72% (metabolism)	GO:0006351, 0.3333±0.4714, 53%<br>(transcription, DNA-dependent)
	GO:0008372 (cellular_component unknown)	n/a	GO:0005622, 0.2276±0.3219, 56%<br>(intracellular)
	GO:0004190 (aspartic-type endopeptidase activity)	GO:0004190, 0.6558±0.4639, 80% (aspartic-type endopeptidase)	n/a
PFB0795w<br>ATP synthase F1, alpha subunit, putative	GO:0000004 (biological process unknown)	GO:0016887, 0.7943±0.3972, 82% (ATPase)<br>GO:0015405, 0.7642±0.3872, 72% (P-P-bond-hydrolysis-driven transporter)	
	GO:0005739 (mitochondrion)	GO:0005754, 0.3108±0.3825, 60%<br>(hydrogen-transporting ATP synthase, catalytic core (sensu Eukarya))	
	GO:00005554 (Molecular function unknown)	GO:0015075, 0.2349±0.3884, 50% (ion transporter)	GO:0016820, 0.7642±0.3872, 62%<br>(hydrolase, acting on acid anhydrides, catalyzing transmembrane movement of substances)<br>GO:0015442, 0.7642±0.3872, 72%<br>(hydrogen-/sodium-translocating ATPase)
